# Poor Response to Gonadotropin Stimulation and Perinatal Outcomes in Fresh In Vitro Fertilization Embryo Transfer Cycles—A Retrospective Cohort Study

**DOI:** 10.3390/jcm13102985

**Published:** 2024-05-19

**Authors:** Alyssa Hochberg, Avital Wertheimer, Rita Zlatkin, Onit Sapir, Eyal Krispin, Tzippy Schohat, Eran Altman, Avi Ben-Haroush, Yoel Shufaro

**Affiliations:** 1 IVF and Infertility Unit, Helen Schneider Hospital for Women, Rabin Medical Center, Petach Tikva 4941492, Israelonitsapir@gmail.com (O.S.); yoel.shufaro@gmail.com (Y.S.); 2 The Faculty of Medicine, Tel Aviv University, Tel Aviv 6997801, Israel

**Keywords:** in vitro fertilization, poor responders, fresh embryo transfer, perinatal outcomes

## Abstract

**Objective:** The objective was to examine the association between poor ovarian response to gonadotropin stimulation for in vitro fertilization (IVF) and adverse perinatal outcomes in singleton gestations in young patients. **Methods:** This was a retrospective cohort study including women aged 17–39 who underwent fresh embryo transfer and delivered a singleton neonate at a single center (pre-implantation genetic testing excluded) (2007–2022). Patients were classified as one of the following categories: poor responders—daily follicle-stimulating hormone (FSH) ≥ 150 IU yielding ≤ 3 retrieved oocytes; normal responders—4–15 oocytes; and high responders with ≥16 oocytes. The primary outcome was a composite of pre-eclampsia (mild or severe), small-for-gestational-age, gestational diabetes mellitus, and preterm birth (<37 weeks). We compared maternal and neonatal outcomes between the three groups. Multivariable logistic regression was used to control for confounders. **Results:** Overall, 507 women met the inclusion criteria. Of them, there were 44 (8.68%) poor responders, 342 (67.46%) normal responders, and 121 (23.87%) high responders. Poor responders, compared to normal and high responders, were characterized by a higher maternal age (34.64 ± 4.01 vs. 31.4 ± 5.04 vs. 30.01 ± 4.93, *p* < 0.001, respectively) and total FSH dosage (3028.41 ± 1792.05 IU vs. 2375.11 ± 1394.05 IU vs. 1869.31 ± 1089.63 IU, *p* < 0.001). The perinatal outcomes examined, including cesarean delivery (CD) rate and the composite outcome, were comparable between groups. Using multivariable logistic regression and adjusting for ovarian response group, maternal age, nulliparity, and estradiol level and endometrial thickness before ovulation triggering, poor response was not associated with CD rate or the composite outcome, with maternal age associated with CD (*p* = 0.005), and nulliparity with the composite outcome (*p* = 0.007). Similar results were obtained when comparing poor responders to each other group separately or to all other responders. **Conclusions:** Poor ovarian response is not associated with increased adverse maternal or neonatal outcomes.

## 1. Introduction

Gestations following assisted reproductive technologies (ART) are associated with a significantly higher risk of adverse perinatal outcomes [1,2], including preterm birth (PTB) and low birthweight (LBW), compared to spontaneous pregnancies, even after adjusting for potential confounders [3,4]. The increasing trend towards single embryo transfer (ET) has resulted in improved obstetric and perinatal outcomes [2]. However, even in singleton pregnancies following in vitro fertilization (IVF), there is still an increased risk of obstetrical complications (hypertensive disorders of pregnancy (HDP), gestational diabetes mellitus (GDM), placenta previa and postpartum hemorrhage (PPH)), as well as perinatal complications (PTB, LBW, small-for-gestational-age (SGA) neonates, and perinatal death), in comparison to spontaneous pregnancies [4,5,6]. This increased risk has been attributed to the underlying infertility itself [4] and to the ovarian stimulation (OS) and embryo culture methods, with specific epigenetic modifications due to IVF techniques [2]. Higher rates of PTB and LBW among women of advanced maternal age (AMA) [7], thought to result from vascular aging and endothelial dysfunction, have also been reported [8]. Vascular endothelial dysfunction associated with AMA is in turn attributed to sex steroid depletion, a consequence of ovarian aging [9]. Therefore, the question arises whether women with a poor response to OS, a manifestation of early ovarian aging, are at increased risk of adverse obstetric outcomes following IVF treatment, regardless of maternal age.

Few published studies have addressed the association between response to gonadotropin stimulation and perinatal outcomes following IVF treatment. It has been demonstrated that women with a poor ovarian response have a poor prognosis, with lower live birth rates (LBRs) [10] and higher miscarriage rates [11]. Additionally, studies analyzing the association between the number of retrieved oocytes and IVF outcomes demonstrated optimal LBRs with 15 oocytes in fresh cycles, with higher numbers associated with an increased risk of ovarian hyperstimulation syndrome and no increase, and even a negative impact, on LBRs [12,13]. However, to date, it is unclear whether the response to OS influences other obstetric and perinatal outcomes following IVF treatment. Theoretically, the obstetric outcomes could be influenced by ovarian and vascular aging in poor responders and by the ovarian dysfunction and probable detrimental effect of very high steroid levels on the endometrium among high responders.

Due to the postulated association between ovarian aging and adverse obstetric and perinatal outcomes, we aimed to evaluate the association between poor ovarian response to gonadotropin stimulation, quantified as the number of oocytes retrieved following stimulation with standard gonadotropin daily dosages, and adverse obstetric and perinatal outcomes following fresh IVF-ET cycles in singleton gestations, in young patients (<40 years of age).

## 2. Materials and Methods

### 2.1. Participants

This was a retrospective cohort study including 507 infertile women aged 17–39 who underwent OS, oocyte pick-up, fresh ET and delivered a singleton neonate at Rabin Medical Center, during 2007–2022. IVF cycles for pre-implantation genetic testing and multiple gestations were excluded. Infertility status was established following 12 or more months of regular unprotected intercourse without conceiving a pregnancy for women below the age of 35 and following 6 months when the female partner was 35 years of age or older, according to the American Society for Reproductive Medicine (ASRM) guidelines’ accepted definition [14]. Infertility etiologies encompassed the following: tubal factor infertility, male factor infertility (defined as a total motile sperm count below 5 million on two semen analyses performed 2–3 months apart), unexplained infertility, polycystic ovary syndrome, and others (including endometriosis and diminished ovarian reserve). Notably, we included women up to the age of 39 inclusive, according to similar definitions of AMA in previous studies. The medical literature is replete with definitions regarding AMA, with many, including those examining reproductive outcomes, focusing on the 39–40 age range [15,16]. Whilst in most countries, the generally accepted definition of AMA is ≥35 years, the age threshold can be raised to 40, 45, or even 50 years when considering age as a risk factor for pregnancy [17]. Additionally, it has been shown that the incidence of adverse pregnancy outcomes is positively related to age [18]. Additionally, we examined only fresh ETs, owing to the low prevalence of surplus embryos for cryopreservation in poor responders, and to better examine the effect of the hormonal milieu on obstetric and perinatal outcomes in the different groups of ovarian response, since a hyper-response is known to significantly increase the risk for ovarian hyperstimulation and thromboembolic events [13,19,20].

### 2.2. Ovarian Stimulation

The fixed or flexible gonadotropin-releasing hormone (GnRH) antagonist protocol and long mid-luteal or short-flare GnRH agonist OS protocols were used in the included cycles. Gonadotropins were given either as recombinant human follicle-stimulating hormone (r-hFSH) (Gonal-F, Merck-Serono, Amsterdam, Netherlands, or Puregon, Organon, New Jersey, United States)), or as combined FSH and luteinizing hormone (LH) products (Menopur, Ferring, Kiel, Germany, or Pergoveris, Merck-Serono, Geneva, Switzerland) at individualized doses according to the patient’s age, weight, ovarian reserve testing, and previous response to OS. The first monitoring visit was scheduled according to the stimulation protocol. The gonadotropin dose could then be adjusted according to the patient’s response as assessed by serum estradiol and progesterone levels, and by follicle number and diameter as assessed by transvaginal ultrasound. Human chorionic gonadotropin (hCG) (250 mg, Ovitrelle, Merck-Serono, Modugno (Bari), Italy), a GnRH agonist (Decapeptyl 0.2 mg, Ferring, Kiel, Germany), or a combination of both was administered for triggering oocyte maturation when at least three follicles reached 17 mm in diameter, and oocyte retrieval was performed 36–38 h later. Intracytoplasmic sperm injection (ICSI), and/or standard IVF were applied as indicated by sperm parameters. Embryos were transferred on day 2–5 of culture under abdominal ultrasound guidance, according to individual patient considerations. Luteal phase support included vaginal progesterone, with the addition of oral estradiol in cases of GnRH agonist-only triggering.

Patients were classified as poor, normal, and high responders. Poor responders were defined as those receiving daily FSH ≥ 150 IU yielding ≤ 3 oocytes, based on standard definitions accepted in clinical practice [21]; high responders as those with ≥16 oocytes retrieved, regardless of FSH dosage [20]; and normal responders as those with 4–15 aspirated oocytes (ranging between a poor and a high response), regardless of FSH dosage. Demographic, clinical, and laboratory cycle outcome parameters, as well as treatment variables on the day of ovulation triggering, were compared between poor, normal, and high responders. The primary outcome was a composite of placental complications, defined as any of the following: preeclampsia; SGA (birthweight below the 10th percentile according to nationally accepted growth curves matched for gestational age (GA) at delivery and fetal sex [22]); GDM; or PTB. We compared adverse obstetric and perinatal outcomes between poor, normal, and high responders.

### 2.3. Data Extraction

Data were retrieved from the comprehensive computerized laboratory and perinatal databases at our center.

### 2.4. Statistics

Statistical analysis was performed using Statistical Analysis System (SAS) Software, Version 9.4 (Cary, NC, USA). Continuous variables were presented as mean (±standard deviation (SD)), and categorical variables as numbers (percentages). The normality of continuous variables was assessed using the Kolmogorov–Smirnov test. For non-normal variables, we used Kruskal–Wallis and Wilcoxon tests. The analysis of variance (ANOVA) test was used to compare continuous variables between the three study groups, and the Chi-squared test and Fisher’s exact test were used to compare categorical variables. The Bonferroni correction was applied as indicated to adjust for multiple comparisons. Multivariable logistic regression was used to model covariate effects on binary outcomes and to adjust for potential confounders. A two-sided *p*-value < 0.05 was considered statistically significant.

### 2.5. Ethics

The study was approved by Rabin Medical Center’s local Institutional Review Board (IRB RMC-19-0697). Informed consent was waived, due to the retrospective design of the study.

## 3. Results

Overall, 507 women met the inclusion criteria. Of them, 44 women (8.68%) were categorized as poor responders, 342 (67.46%) as normal responders, and 121 (23.87%) as high responders. Baseline maternal and cycle characteristics of the study cohort are presented in Table 1 and Table 2, respectively. Notably, to adjust for multiple comparisons, the Bonferroni correction was applied, such that statistically significant differences between groups were those with a *p*-value < 0.01 in Table 1 and *p* < 0.004 in Table 2. Poor responders, compared to normal and high responders, were characterized by an increased maternal age, and increased total and daily FSH dosages. Notably, daily FSH doses ranged from 150–600 IU in the poor responder group, 66–600 IU in the normal responder group, and 75–450 IU in the high responder group. Unsurprisingly, poor responders had fewer retrieved oocytes, transferred embryos, frozen embryos, blastocyst transfers, and gonadotropin stimulation days, as well as lower pre-trigger estradiol and progesterone levels (Table 2). Cycle number, infertility cause, primary or secondary infertility rates, gravidity, and parity were all comparable between groups (Table 1).

Table 3 demonstrates the obstetric and perinatal outcomes of the three groups. Notably, after applying the Bonferroni correction adjusting for multiple comparisons, a *p*-value of *p* < 0.002 was considered statistically significant. As depicted in the table, there were no between-group differences in GA at delivery, rate of cesarean delivery (CD), birthweight, and the rates of preeclampsia, GDM, PPH, placental abruption, large-for-gestational-age neonates (defined as birthweight above the 90th percentile according to nationally accepted growth curves matched for GA at delivery and fetal sex [22]), SGA, or the composite outcome.

Using multivariable logistic regression, controlling for ovarian response group, maternal age, maximal estradiol levels prior to ovulation triggering, endometrial thickness prior to ovulation triggering, and nulliparity, poor ovarian response was not found to be associated with CD rate when compared to normal and high responders (adjusted odds ratio (aOR) 1.19, 95% confidence interval (CI) 0.6–2.37, *p* = 0.614; aOR 1.53, 95% CI 0.67–3.5, *p* = 0.313, respectively) (Table 4). However, increasing maternal age was found to increase the odds for CD (aOR 1.06, 95% CI 1.02–1.11, *p* = 0.005).

Additionally, in the multivariable logistic regression adjusting for the variables mentioned above, poor ovarian response, compared to a normal and high response, was not associated with the composite outcome (aOR 0.9, 95% CI 0.43–1.86, *p* = 0.768; aOR 1.42, 95% CI 0.6–3.35, *p* = 0.430, respectively) (Table 5). The only parameter found to be associated with the composite outcome was nulliparity (aOR 1.75, 95% CI 1.17–2.63, *p* = 0.007, Table 4), which increased the risk for the composite outcome. Notably, poor ovarian response was not found to be associated with CD rate or the composite outcome when compared to normal and high responders separately or together.

## 4. Discussion

We compared the occurrence of adverse obstetric and perinatal outcomes between poor, normal, and high responders, grouped according to the number of oocytes retrieved after OS, following fresh IVF-ET cycles in singleton gestations. Our key findings were as follows: (1) Poor responders did not differ in perinatal outcomes or the composite outcome compared to the other groups. (2) Increasing maternal age increased the odds for CD. (3) The only parameter associated with the composite outcome was nulliparity, which increased the odds for the composite outcome.

Whilst it has been demonstrated that women with a poor ovarian response have a poorer reproductive prognosis compared to other patient groups, with lower LBRs [10] and higher miscarriage rates [11], few studies, with contradictory results, have investigated the association between the number of oocytes retrieved during OS for ART and adverse obstetric and perinatal outcomes. Additionally, many of these studies did not consider the daily FSH dosage given in a fresh cycle when defining a group of poor responders, versus simply those given inadequate dosages. Numerous studies address the association between high ovarian response to stimulation (>20 retrieved oocytes) and adverse perinatal outcomes, possibly due to various concerns regarding this group. For instance, OS for IVF resulting in a high serum estradiol peak on the day of hCG administration, as opposed to a more moderate rise in estradiol, has been suggested as a risk factor for LBW [23]. Additionally, a large retrospective study from the United Kingdom, including more than 65,000 singleton births after fresh IVF-ET, found an association between excessive ovarian response (>20 oocytes) and an increased risk of PTB and LBW, compared with women with a normal response (10–15 oocytes) [24]. Similarly to the results in our study, they found no increased risk for the aforementioned adverse outcomes among women with a poor ovarian response (≤three oocytes) compared with normal responders, though they did not address gonadotropin dosages given to supposed “poor responders”, as we did. As opposed to that study, another retrospective study including 8941 IVF singletons born after fresh treatment cycles, investigating factors affecting obstetric outcome after IVF, found no association between very LBW or SGA and the number of oocytes retrieved [3]. Notably, one must acknowledge that normal and high responders in our study did not all receive daily FSH doses at and above 150 IU. However, since poor responders are usually administered increased FSH doses (with a minimal required dose to designate them as true poor responders), and high responders are administered lower doses, daily FSH dose essentially constitutes an inherent part of correct patient categorization. Therefore, daily FSH dose cannot be properly adjusted for in a model regarding its potential effect on perinatal outcomes. However, our group designations into poor, normal, and high responders, aligning with the group definitions accepted in the literature and in clinical practice, add to the validity of our findings regarding the effect of ovarian stimulation response groups on perinatal outcomes.

A retrospective cohort study by Richardson et al. [25], specifically addressing poor ovarian reserve, investigated whether a poor ovarian reserve test influences perinatal outcomes independently of age. They included fresh single ET IVF/ICSI cycles and defined ovarian reserve according to anti-Müllerian hormone (AMH) levels, with an AMH ≤ 5.4 pmol/L considered poor and 5.41–24.99 pmol/L considered normal. In their cohort of 1520 women, they found no significant differences in rates of congenital anomalies, birthweight, or GA at delivery between groups after adjusting for maternal age. However, this study, as opposed to ours, defined poor ovarian reserve based on pre-treatment AMH levels and not by the actual response to gonadotropin stimulation, possibly representing a different patient population.

Another retrospective study including 27,359 singleton live births resulting from fresh IVF-ET cycles examined the association between the number of oocytes retrieved for IVF and perinatal outcomes [26]. No significant association was observed between the number of oocytes retrieved (continuous variable) and PTB (<37 weeks of gestation), very PTB (<32 weeks of gestation), SGA (<2SD), HDP, peri/neonatal death, or major birth defects. However, a significant association was found for placenta previa with increasing number of oocytes retrieved, and for the secondary outcome variable gender distribution, with a higher rate of males after retrieval of >20 oocytes. As in our study, no significant associations were found between poor responders (≤3 oocytes) and any obstetric outcomes investigated. However, this group was compared only to a limited reference group in which slightly more (four–nine) oocytes were retrieved, and not to patients with a substantially higher number of aspirated oocytes, whether considered in the range of normal or high ovarian response, as was carried out in our study. Additionally, poor responders were defined according to the number of retrieved oocytes, without considering gonadotropin daily dosages, not separating those sub-optimally stimulated from true poor responders. Another matched controlled study comparing pregnancy outcomes following IVF in 150 women with a poor ovarian response (≤three oocytes retrieved after receiving ≥150 IU of daily FSH) and 150 women with a normal response (eight–twelve oocytes retrieved) found no significant differences in the incidences of HDP, GA at delivery, and birthweight [27], similar to our findings. However, they did not compare this group to additional assumed normal responders with four–seven oocytes retrieved, nor to a group of high responders, as we did.

Regarding the association between increasing maternal age and increased odds for CD, this finding is in accordance with reported literature, with a higher CD rate with AMA (historically defined as ≥35 years) [28].

Similar to previous studies, we found nulliparity to be associated with increased odds for the composite outcome, possibly due to its well-established association with an increased risk for preeclampsia [29], SGA [30], GDM [31], and PTB [32], all of which were components of the composite outcome.

The strength of our study lies in its single-center design, with standardized clinical treatment and laboratory management protocols, as well as obstetrical care and delivery. Additionally, our cohort included only relatively young patients < 40 years of age, in order to more specifically examine ovarian aging in this patient population. We also strictly defined the poor responder group as those receiving daily FSH ≥ 150IU yielding ≤ three oocytes, thus allowing for a more accurate and contemporary definition of this patient group than in previously published studies. Furthermore, we believe the comparison between poor responders and normal and high responders as separate groups allowed for more precise examination of potential perinatal complications unique to this specific sub-group.

The main limitation of our study lies in its retrospective nature and the limited sample size of poor responders. Notably, our cohort consisted of a relatively small number of poor responders due to our unit being a large referral center for IVF treatment, surrounded by many hospitals that provide birthing and delivery care; therefore, whilst many poor responders received IVF treatment at our facility during the study period, a much smaller number went on to have a live birth and delivery at our institution. Nonetheless, restricting our cohort to patients who underwent IVF treatment and delivered at a single center allowed for better standardization of diagnoses and care, thus optimizing conditions for the three-group comparison. Additionally, we did not have data regarding patients’ body mass index (BMI) or medication use (such as aspirin), variables that may affect the risk for obstetrical complications [33,34]. Nonetheless, it should be noted that the BMI cut-off for IVF treatment at our institution is <35 kg/m^2^. Furthermore, as previously mentioned, due to FSH daily dose constituting an inherent characteristic of the study groups, we could not adjust for it in a multivariate logistic regression model, and therefore could not tease out the pure effect of FSH dose on perinatal outcomes. Lastly, it is worth mentioning that part of our study period (2019–2022) overlapped with the coronavirus disease 2019 (COVID-19) pandemic. Though COVID-19 infection status was not assessed in our study, previous studies have indicated no statistically significant difference between COVID-19 positive and COVID-19 negative patients regarding pregnancy outcomes (such as neonatal intensive care unit admission and stillbirth) [35,36], while others suggest an association between specific COVID-19 variants and various pregnancy complications (such as PTB) [37].

## 5. Conclusions

We found that poor ovarian response to conventional OS in patients < 40 years of age was not associated with a higher rate of adverse obstetric or perinatal outcomes, despite this group’s acknowledged ovarian aging. Notwithstanding our study’s retrospective nature and small sample size, we believe these findings may reassure physicians and patients that, although chances for pregnancy and live birth are decreased, once pregnant, these patients are not prone to increased obstetric complications, and hence do not require special management and follow-up if no other risk factors for obstetric complications exist. Future large prospective studies are needed to corroborate our findings.

## Figures and Tables

**Table 1:** Baseline maternal characteristics.

Variable	Poor Responders (N = 44)	Normal Responders (N = 342)	High Responders (N = 121)	*p*-Value
Age (years)	34.64 ± 4.01	31.4 ± 5.04	30.01 ± 4.93	**<0.001**
Infertility cause (%)				0.274
Tubal factor	6 (13.64)	34 (9.94)	9 (7.44)	
Male factor	13 (29.55)	138 (40.35)	64 (52.89)	
Unexplained	11 (25)	78 (22.81)	21 (17.36)	
PCOS ^a^	1 (2.27)	18 (5.26)	12 (9.92)	
Other ^b^	13 (29.55)	74 (21.637)	15 (12.397)	
Infertility (%)				0.703
Primary	16 (36.36)	140 (40.94)	45 (37.19)	
Secondary	28 (63.64)	201 (58.77)	75 (61.98)	
Unknown	0 (0)	1 (0.29)	1 (0.83)	
Gravidity (G) (%)				0.83
0	17 (38.64)	139 (40.64)	46 (38.02)	
1+	27 (61.36)	200 (58.48)	75 (61.98)	
Unknown	0 (0)	3 (0.877)	0 (0)	
Parity (P) (%)				0.917
0	24 (54.55)	191 (55.85)	65 (53.72)	
1+	20 (45.45)	151 (44.15)	56 (46.28)	

Continuous variables are presented as mean (± SD), and categorical variables are presented as n (%). ^a^ PCOS—polycystic ovary syndrome; ^b^ other—including endometriosis and diminished ovarian reserve. Significant differences (*p* < 0.01) are presented in bold.

**Table 2:** In vitro fertilization cycle characteristics.

Variable	Poor Responders (N = 44)	Normal Responders (N = 342)	High Responders (N = 121)	*p*-Value
Cycle number	3.34 ± 2.61	3.04 ± 2.71	2.80 ± 2.27	0.488
Protocol (%)				0.034
Agonist	4 (9.09)	75 (21.93)	35 (28.93)	
Antagonist	33 (75)	186 (54.39)	56 (46.28)	
Other	7 (15.9)	81 (23.684)	30 (24.793)	
Daily FSH ^a^ dosage	352.58 ± 142.71	249.60 ± 192.04	188.07 ± 89.91	**<0.001**
Number of gonadotropin stimulation days	8.34 ± 2.76	9.89 ± 2.94	10.09 ± 4.05	**0.003**
Total FSH ^a^ dosage (IU)	3028.41 ± 1792.05	2375.11 ± 1394.05	1869.31 ± 1089.63	**<0.001**
Estradiol before triggering (pmol/L)	2837.32 ± 1592.47	5648.49 ± 2954.97	7537.29 ± 3615.73	**<0.001**
Progesterone before triggering (nmol/L)	1.43 ± 0.85	2.09 ± 2.07	2.27 ± 1.43	**<0.001**
Endometrial thickness (mm) before triggering	9.78 ± 2.78	10.57 ± 2.32	10.57 ± 2.60	0.033
Number of oocytes retrieved	2.41 ± 0.79	9.31 ± 3.34	20.21 ± 5.40	**<0.001**
Fertilization (%)				0.008
IVF ^b^	15 (34.09)	80 (23.39)	19 (15.70)	
ICSI ^c^	28 (63.64)	205 (59.94)	79 (65.29)	
IVF + ICSI	0 (0)	57 (16.67)	23 (19.01)	
Unknown	1 (2.4)	0 (0)	0 (0)	
Number of embryos transferred	1.57 ± 0.62	2.09 ± 0.70	1.98 ± 0.63	**<0.001**
Blastocyst transfer	0 (0)	12 (3.51)	13 (10.74)	**0.002**
Number of frozen embryos	0.07 ± 0.33	1.49 ± 1.94	4.63 ± 4.09	**<0.001**

Continuous variables are presented as mean (± SD), and categorical variables are presented as n (%). ^a^ FSH—follicle stimulating hormone; ^b^ IVF—in vitro fertilization; ^c^ ICSI—intracytoplasmic sperm injection. Significant differences (*p* < 0.004) are presented in bold.

**Table 3:** Obstetric and perinatal outcomes.

Variable	Poor Responders (N = 44)	Normal Responders (N = 342)	High Responders (N = 121)	*p*-Value
Gestational age at delivery (week)	38.4 ± 1.50	38.50 ± 2.06	38.64 ± 1.84	0.501
Mode of delivery:				0.036 *
NVD ^a^ (%)	18 (40.91)	207 (60.53)	75 (61.98)	
Instrumental delivery ^b^ (%)	8 (18.18)	33 (9.65)	18 (14.88)	
CD ^c^ (%)	18 (40.91)	102 (29.82)	28 (23.14)	
Pregnancy-induced hypertension (%)	1 (2.27)	3 (0.88)	2 (1.65)	0.631
Chronic hypertension (%)	1 (2.27)	10 (2.92)	0 (0)	0.165
Any hypertension ^d^ (%)	2 (4.55)	15 (4.39)	5 (4.13)	0.992
Mild preeclampsia (%)	2 (4.55)	10 (2.92)	1 (0.83)	0.321
Severe preeclampsia (%)	1 (2.27)	2 (0.58)	0 (0)	0.252
Gestational diabetes mellitus (%)	3 (6.82)	36 (10.53)	9 (7.44)	0.486
Any diabetes mellitus (%)	2 (4.55)	37 (10.82)	10 (8.26)	0.332
Oligohydramnios (%)	5 (11.36)	14 (4.09)	4 (3.31)	0.079
Polyhydramnios (%)	0 (0)	9 (2.63)	2 (1.65)	0.473
3rd or 4th degree perineal tears (%)	0 (0)	3 (0.88)	0 (0)	0.482
Post-partum hemorrhage (%)	0 (0)	10 (2.92)	6 (4.96)	0.244
Blood products transfusion (%)	0 (0)	2 (0.58)	3 (2.48)	0.150
Maternal fever (%)	2 (4.55)	8 (2.34)	4 (3.31)	0.658
Shoulder dystocia (%)	0 (0)	1 (0.29)	0 (0)	0.784
Placental abruption (%)	1 (2.27)	6 (1.75)	0 (0)	0.32
5 min Apgar score				0.506
≤7 (%)	1 (2.27)	9 (2.63)	1 (0.83)	
>7 (%)	43 (97.73)	332 (97.08)	119 (98.35)	
Not documented (%)	0 (0)	1 (0.29)	1 (0.83)	
Birthweight (grams)	3097.86 ± 505.19	3071.30 ± 541.54	3116.18 ± 555.52	0.823
Birthweight percentile	52.64 ± 29.01	49.31 ± 28.82	50.48 ± 27.51	0.775
Small-for-gestational-age neonates (%)	3 (6.82)	30 (8.77)	5 (4.13)	0.250
Large-for-gestational-age neonates (%)	4 (9.09)	28 (8.19)	9 (7.44)	0.941
Composite outcome ^e^ (%)	13 (29.55)	111 (32.46)	29 (23.97)	0.216

Continuous variables are presented as mean (± SD), and categorical variables are presented as n (%). * Overall *p*-value comparing the 3 study groups. Additionally, no significant differences were found between poor and high responders, normal and high responders, or poor and normal responders (*p* > 0.002). ^a^ NVD—normal vaginal delivery; ^b^ instrumental delivery—vacuum extraction or forceps delivery; ^c^ CD—cesarean delivery; ^d^ any hypertension—including gestational hypertension and chronic hypertension; ^e^ composite outcome—any one of the following: preeclampsia (mild or severe), small-for-gestational-age, gestational diabetes, or preterm birth (<37 weeks).

**Table 4:** Multivariable logistic regression of factors associated with cesarean delivery rate.

Variable	aOR (95% CI)	*p*-Value
Poor responders vs. normal responders	1.19 (0.6–2.37)	0.614
Poor responders vs. high responders	1.53 (0.67–3.5)	0.313
Poor responders vs. normal/high responders	1.22 (0.62–2.43)	0.563
Maternal age	1.06 (1.02–1.11)	**0.005**
Estradiol level prior to ovulation triggering (pmol/L)	1 (1–1)	0.368
Endometrial thickness prior to ovulation triggering (mm)	0.95 (0.88–1.03)	0.234
Nulliparity	0.95 (0.63–1.42)	0.791

Abbreviations: aOR—adjusted odds ratio; CI—confidence interval. Significant differences (*p* < 0.05) are presented in bold.

**Table 5:** Multivariable logistic regression of factors associated with the composite outcome ^a^.

Variable	aOR (95% CI)	*p*-Value
Poor responders vs. normal responders	0.9 (0.43–1.86)	0.768
Poor responders vs. high responders	1.42 (0.6–3.35)	0.430
Poor responders vs. normal/high responders	0.94 (0.45–1.95)	0.864
Maternal age	0.996 (0.96–1.04)	0.862
Estradiol level prior to ovulation triggering (pmol/L)	1 (1–1)	0.476
Endometrial thickness prior to ovulation triggering (mm)	0.93 (0.85–1.01)	0.068
Nulliparity	1.75 (1.17–2.63)	**0.007**

^a^ Composite outcome—any of the following: pre-eclampsia (mild or severe), gestational diabetes, small-for-gestational-age, and preterm birth (<37 weeks). Abbreviations: aOR—adjusted odds ratio; CI—confidence interval. Significant differences (*p* < 0.05) are presented in bold.

## Data Availability

Dataset available on request from the authors.

## Submission Declaration

The material contained in the manuscript was published after partial analysis and presented as a poster presentation at the 41st Virtual Annual Pregnancy Meeting of the Society for Maternal–Fetal Medicine (SMFM), held on 19–30 January 2021. However, the final results after analysis have not been published until now and are solely submitted for consideration for publication in the *Journal of Clinical Medicine*.

## References

[1] McDonald S.D., Han Z., Mulla S., Murphy K.E., Beyene J., Ohlsson A. (2009). Preterm birth and low birth weight among in vitro fertilization singletons: A systematic review and meta-analyses. Eur. J. Obstet. Gynecol. Reprod. Biol..

[2] Pinborg A., Wennerholm U.B., Romundstad L.B., Loft A., Aittomaki K., Söderström-Anttila V., Nygren K.G., Hazekamp J., Bergh C. (2013). Why do singletons conceived after assisted reproduction technology have adverse perinatal outcome? Systematic review and meta-analysis. Hum. Reprod. Update.

[3] Sazonova A., Källen K., Thurin-Kjellberg A., Wennerholm U.-B., Bergh C. (2011). Factors affecting obstetric outcome of singletons born after IVF. Hum. Reprod..

[4] Luke B., Gopal D., Cabral H., Stern J.E., Diop H. (2017). Pregnancy, birth, and infant outcomes by maternal fertility status: The Massachusetts Outcomes Study of Assisted Reproductive Technology. Am. J. Obstet. Gynecol..

[5] Wennerholm U.B., Henningsen A.K., Romundstad L.B., Bergh C., Pinborg A., Skjaerven R., Forman J., Gissler M., Nygren K.G., Tiitinen A. (2013). Perinatal outcomes of children born after frozen-thawed embryo transfer: A Nordic cohort study from the CoNARTaS group. Hum. Reprod..

[6] Qin J., Liu X., Sheng X., Wang H., Gao S. (2016). Assisted reproductive technology and the risk of pregnancy-related complications and adverse pregnancy outcomes in singleton pregnancies: A meta-analysis of cohort studies. Fertil. Steril..

[7] Ludford I., Scheil W., Tucker G., Grivell R. (2012). Pregnancy outcomes for nulliparous women of advanced maternal age in South Australia, 1998–2008. Aust. N. Z. J. Obstet. Gynaecol..

[8] Bonamy A.K.E., Parikh N.I., Cnattingius S., Ludvigsson J.F., Ingelsson E. (2011). Birth characteristics and subsequent risks of maternal cardiovascular disease: Effects of gestational age and fetal growth. Circulation.

[9] Vita J.A., Keaney J.F. (2001). Hormone replacement therapy and endothelial function: The exception that proves the rule?. Arterioscler. Thromb. Vasc. Biol..

[10] Ulug U., Ben-Shlomo I., Turan E., Erden H.F., Akman M.A., Bahceci M. (2003). Conception rates following assisted reproduction in poor responder patients: A retrospective study in 300 consecutive cycles. Reprod. Biomed. Online.

[11] Sunkara S.K., Khalaf Y., Maheshwari A., Seed P., Coomarasamy A. (2014). Association between response to ovarian stimulation and miscarriage following IVF: An analysis of 124 351 IVF pregnancies. Hum. Reprod..

[12] Sunkara S.K., Rittenberg V., Raine-Fenning N., Bhattacharya S., Zamora J., Coomarasamy A. (2011). Association between the number of eggs and live birth in IVF treatment: An analysis of 400 135 treatment cycles. Hum. Reprod..

[13] Steward R.G., Lan L., Shah A.A., Yeh J.S., Price T.M., Goldfarb J.M., Muasher S.J. (2014). Oocyte number as a predictor for ovarian hyperstimulation syndrome and live birth: An analysis of 256,381 in vitro fertilization cycles. Fertil. Steril..

[14] (2023). Definition of Infertility: A Committee Opinion|American Society for Reproductive Medicine|ASRM [Internet]. https://www.asrm.org/practice-guidance/practice-committee-documents/denitions-of-infertility/.

[15] Haas J., Miller T.E., Nahum R., Aizer A., Kirshenbaum M., Zilberberg E., Lebovitz O., Orvieto R. (2021). The role of ICSI vs. conventional IVF for patients with advanced maternal age-a randomized controlled trial. J. Assist. Reprod. Genet..

[16] Tannus S., Son W.-Y., Gilman A., Younes G., Shavit T., Dahan M.-H. (2017). The role of intracytoplasmic sperm injection in non-male factor infertility in advanced maternal age. Hum. Reprod..

[17] Zhang C., Yan L., Qiao J. (2022). Effect of advanced parental age on pregnancy outcome and offspring health. J. Assist. Reprod. Genet..

[18] Zhu C., Wang M., Niu G., Yang J., Wang Z. (2018). Obstetric outcomes of twin pregnancies at advanced maternal age: A retrospective study. Taiwan. J. Obstet. Gynecol..

[19] Magnusson Å., Källen K., Thurin-Kjellberg A., Bergh C. (2018). The number of oocytes retrieved during IVF: A balance between efficacy and safety. Hum. Reprod..

[20] Drakopoulos P., Khalaf Y., Esteves S.C., Polyzos N.P., Sunkara S.K., Shapiro D., Rizk B., Ye H., Costello M., Koloda Y. (2023). Treatment algorithms for high responders: What we can learn from randomized controlled trials, real-world data and models. Best Pract. Res. Clin. Obstet. Gynaecol..

[21] Ferraretti A.P., La Marca A., Fauser B.C.J.M., Tarlatzis B., Nargund G., Gianaroli L. (2011). ESHRE consensus on the definition of “poor response” to ovarian stimulation for in vitro fertilization: The Bologna criteria. Hum. Reprod..

[22] Dollberg S., Haklai Z., Mimouni F.B., Gorfein I., Gordon E.S. (2005). Birth weight standards in the live-born population in Israel. Isr. Med. Assoc. J..

[23] Pereira N., Reichman D.E., Goldschlag D.E., Lekovich J.P., Rosenwaks Z. (2015). Impact of elevated peak serum estradiol levels during controlled ovarian hyperstimulation on the birth weight of term singletons from fresh IVF-ET cycles. J. Assist. Reprod. Genet..

[24] Sunkara S.K., La Marca A., Seed P.T., Khalaf Y. (2015). Increased risk of preterm birth and low birthweight with very high number of oocytes following IVF: An analysis of 65,868 singleton live birth outcomes. Hum. Reprod..

[25] Richardson A., Mascarenhas M., Balen A. (2021). Is a woman’s chronological age or “ovarian age” more important in determining perinatal outcome after assisted reproductive treatment?. Hum. Fertil..

[26] Magnusson Å., Wennerholm U.-B., Källén K., Petzold M., Thurin-Kjellberg A., Bergh C. (2018). The association between the number of oocytes retrieved for IVF, perinatal outcome and obstetric complications. Hum. Reprod..

[27] van Disseldorp J., Eijkemans R., Fauser B., Broekmans F. (2010). Hypertensive pregnancy complications in poor and normal responders after in vitro fertilization. Fertil. Steril..

[28] Ayrampour H., Heaman M. (2010). Advanced maternal age and the risk of cesarean birth: A systematic review. Birth.

[29] Overview|Antenatal Care|Guidance|NICE. https://www.nice.org.uk/guidance/ng201.

[30] Desplanches T., Bouit C., Cottenet J., Szczepanski E., Quantin C., Fauque P., Sagot P. (2019). Combined effects of increasing maternal age and nulliparity on hypertensive disorders of pregnancy and small for gestational age. Pregnancy Hypertens..

[31] Schneider S., Freerksen N., Röhrig S., Hoeft B., Maul H. (2012). Gestational diabetes and preeclampsia--similar risk factor profiles?. Early Hum. Dev..

[32] Koullali B., van Zijl M.D., Kazemier B.M., Oudijk M.A., Mol B.W.J., Pajkrt E., Ravelli A.C.J. (2020). The association between parity and spontaneous preterm birth: A population based study. BMC Pregnancy Childbirth.

[33] Falcone V., Stopp T., Feichtinger M., Kiss H., Eppel W., Husslein P.W., Prager G., Göbl C.S. (2018). Pregnancy after bariatric surgery: A narrative literature review and discussion of impact on pregnancy management and outcome. BMC Pregnancy Childbirth.

[34] Rolnik D.L., Wright D., Poon L.C.Y., Syngelaki A., O’Gorman N., de Paco Matallana C., Akolekar R., Cicero S., Janga D., Singh M. (2017). ASPRE trial: Performance of screening for preterm pre-eclampsia. Ultrasound Obstet. Gynecol.

[35] Adam A.-M., Popa R.-F., Vaduva C., Georgescu C.V., Adam G., Melinte-Popescu A.-S., Popa C., Socolov D., Nechita A., Vasilache I.-A. (2023). Pregnancy Outcomes, Immunophenotyping and Immunohistochemical Findings in a Cohort of Pregnant Patients with COVID-19-A Prospective Study. Diagnostics.

[36] Wilkinson M., Johnstone E.D., Simcox L.E., Myers J.E. (2022). The impact of COVID-19 on pregnancy outcomes in a diverse cohort in England. Sci. Rep..

[37] Stock S.J., Moore E., Calvert C., Carruthers J., Denny C., Donaghy J., Hillman S., Hopcroft L.E., Hopkins L., Goulding A. (2022). Pregnancy outcomes after SARS-CoV-2 infection in periods dominated by delta and omicron variants in Scotland: A population-based cohort study. Lancet Respir. Med..

